# Chronic granulomatous disease mimicking early-onset Crohn’s disease with cutaneous manifestations

**DOI:** 10.1186/1471-2431-14-156

**Published:** 2014-06-20

**Authors:** Maria Barbato, Giovanni Ragusa, Fortunata Civitelli, Adriana Marcheggiano, Giovanni Di Nardo, Metello Iacobini, Taulant Melengu, Salvatore Cucchiara, Marzia Duse

**Affiliations:** 1 Pediatric Gastroenterology, Endoscopy and Liver Unit Sapienza University of Rome, Rome, Italy; 2 Pediatric Immunology Unit, Sapienza University of Rome, Viale Regina Elena, 324-00161 Rome, Italy; 3 Department of Clinical Sciences – Gastroenterology Unit, Sapienza University of Rome, Rome, Italy; 4 Pediatric Hematology Unit, Sapienza University of Rome, Rome, Italy

**Keywords:** Chronic granulomatous disease, Crohn’s disease, Serratia marcescens, Celiac disease, Skin infection, Nitroblue tetrazolium test, Pigment laden histiocytes

## Abstract

**Background:**

Chronic granulomatous disease is a rare inherited disorder of the innate immune system. In patients with a clinical history of recurrent or persistent infections, especially infections caused by uncommon species, chronic granulomatous disease should be considered.

**Case presentation:**

We report the case of a 5-year-old boy with a presumptive diagnosis of Crohn’s disease with extraintestinal manifestations. Chronic granulomatous disease was suspected in this case after *Serratia marcescens* was isolated from a skin ulcer culture. Granulomas were confirmed on histology and chronic granulomatous disease was diagnosed.

**Conclusion:**

This case emphasizes the importance of high clinical suspicion of an alternative diagnosis of immune deficiency in patients with presumed inflammatory bowel disease and opportunistic infections, especially when disease occurs in early life.

## Background

Chronic granulomatous disease (CGD) is a rare inherited disorder of the innate immune system caused by mutations in any of the genes encoding subunits of the superoxide-generating phagocyte NADPH oxidase, which is essential for killing catalase-producing bacteria and fungi [1]. In patients with a history of recurrent or persistent infections, particularly infections caused by uncommon species such as *Aspergillus*, *Staphylococcus aureus*, *Serratia marcescens*, *Nocardia,* and *Burkholderia cepacia*, CGD should be considered [2]. A hallmark of CGD is an abnormal inflammatory response leading to the formation of granulomas in multiple tissues, both in the presence and absence of microorganisms. We report the case of a 5-year-old boy who had an initial clinical presentation mimicking Crohn’s disease with cutaneous manifestations and multiple granulomas on intestinal biopsies, whose diagnosis ultimately proved to be CGD. Culture of the patient’s ulcerated skin lesions revealed *Serratia* infection, which was successfully treated with itraconazole and cotrimoxazole.

## Case presentation

**Figure 1:**
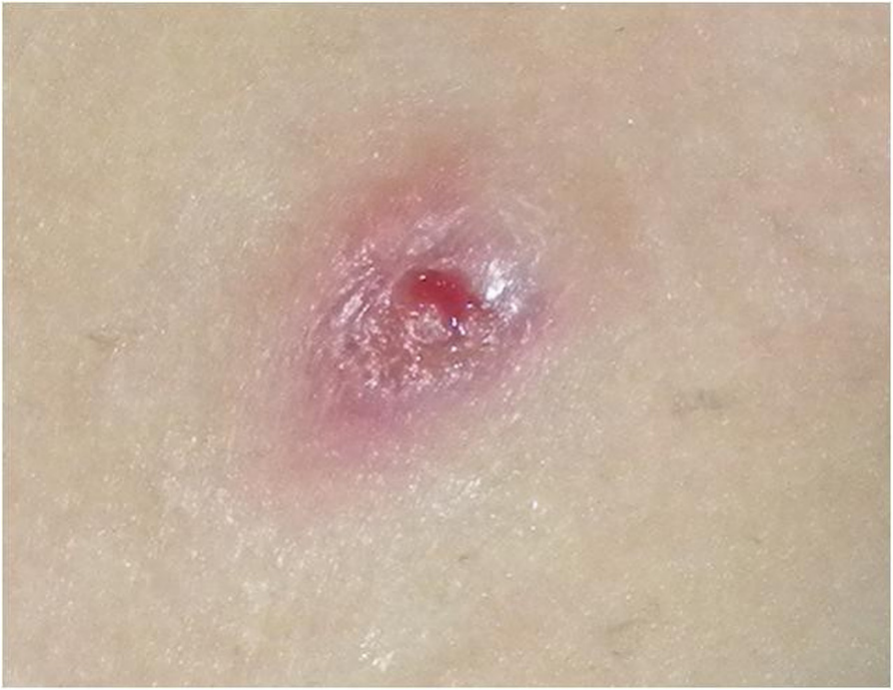
Demarcated ulcers with violaceous edges and a yellow surface.

A 5-year-old Italian boy was admitted to our Pediatric Gastroenterology Unit with a presumptive diagnosis of metastatic Crohn’s disease (CD) or cutaneous manifestations of inflammatory bowel disease.The patient’s past medical history included neonatal pyoderma requiring antibiotic therapy, intermittent diarrhea and abdominal pain since 2 years of age, and hospitalization at the age of three for treatment of severe pneumonia with pleural effusion. Laboratory tests at time of hospitalization showed elevated inflammatory parameters, iron deficiency anemia, and positive serologic screening for celiac disease, later confirmed by endoscopy. The patient started a gluten-free diet but continued to have sporadic episodes of diarrhea and abdominal pain, recurrent oral ulcerations, persistent anemia, and elevated inflammatory markers. The following year, the patient experienced a relapse of pneumonia, preceded 7 days earlier by fever, abdominal pain, and diarrhea, followed by the appearance of two painful red nodules, one on the left leg and one on the lower left abdominal region. The lesions progressively evolved into round demarcated ulcers with violaceous edges and a yellow surface (Figure 1).

Physical examination at presentation showed poor general condition and nutritional status, mild cervical lymphadenopathy, tenderness of the lower left abdomen during examination, and perianal skin tags, but no hepatosplenomegaly or other masses. Physical examination revealed that the patient’s height and weight were below the 10th percentile.

**Figure 2:**
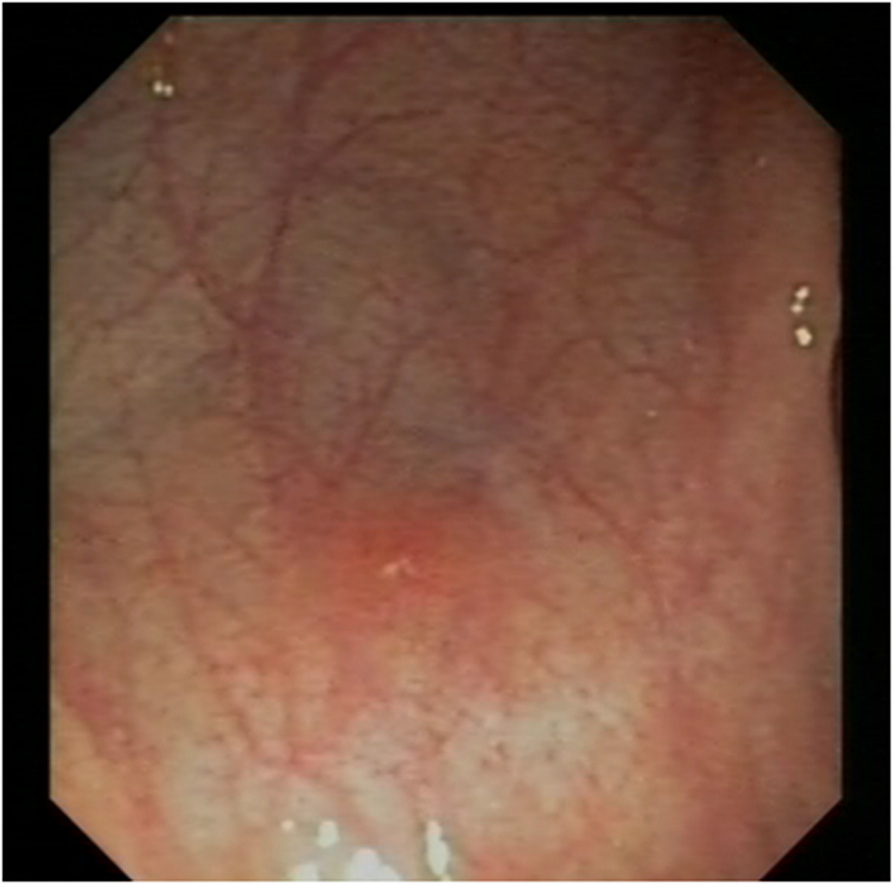
Isolated aphthous ulcerations in the left colon with normal mucosa.

Laboratory tests demonstrated leukocytosis (WBC 20,500/μL, 65% neutrophils, 19%, lymphocytes, 5% monocytes, 9% eosinophils), thrombocytosis (PLT 635,000/μL), iron deficiency anemia, hypoalbuminemia, and elevated fecal calprotectin level. Inflammatory bowel disease markers were positive for IgA and IgG antibodies against *Saccharomyces cerevisiae* (ASCA) and negative for perinuclear anti-nuclear cytoplasmic antibodies. Topical and systemic antibiotic therapy had been performed without success.We performed ileocolonoscopy, which revealed isolated aphthous ulcerations in the left colon with normal mucosa (Figure 2). Histology showed mild distortion of crypt architecture, cryptitis, and the presence of epithelioid granulomas in the lamina propria of both the ileum and the colon, a picture consistent with CD. Skin biopsies documented neutrophilic vasculitis consistent with pyoderma gangrenosum, and lesions were thus interpreted as an extraintestinal manifestation of CD.

Culture of cutaneous lesions grew *Serratia marcescens*, a catalase positive, Gram-negative bacillus. Isolated *Serratia* infections are rare in children, but they are a common feature of primary immunodeficiency, particularly of phagocytic dysfunction disorders such as CGD [3]. The patient was referred to our Immunology Unit for evaluation and further diagnostic work-up. Repeat intestinal biopsies were also required. The nitroblue tetrazolium test, used to investigate phagocytic function, revealed a complete inability to produce a normal respiratory burst, supporting the suspected diagnosis of CGD.

**Figure 3:**
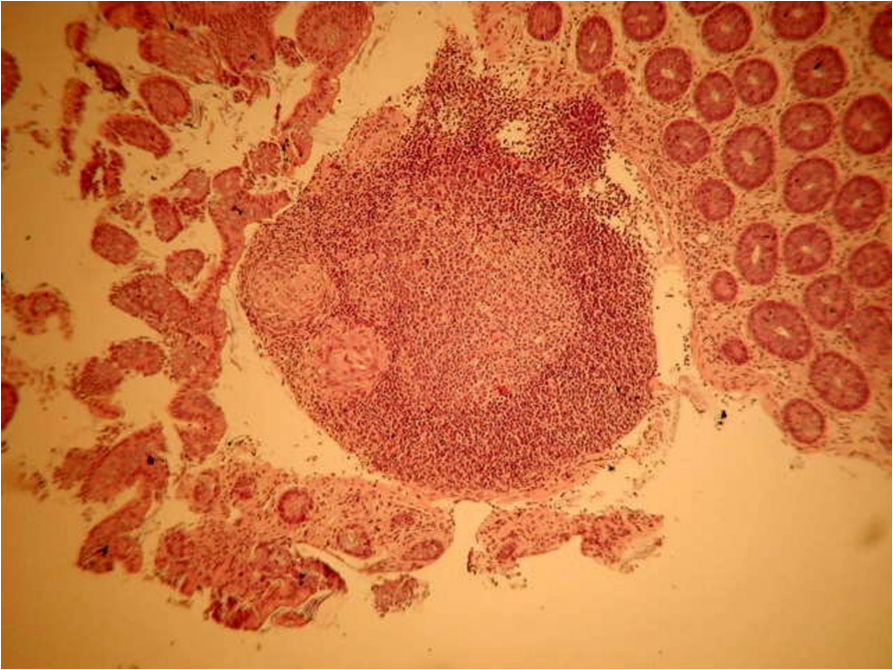
Granulomas within the lamina propria.

**Figure 4:**
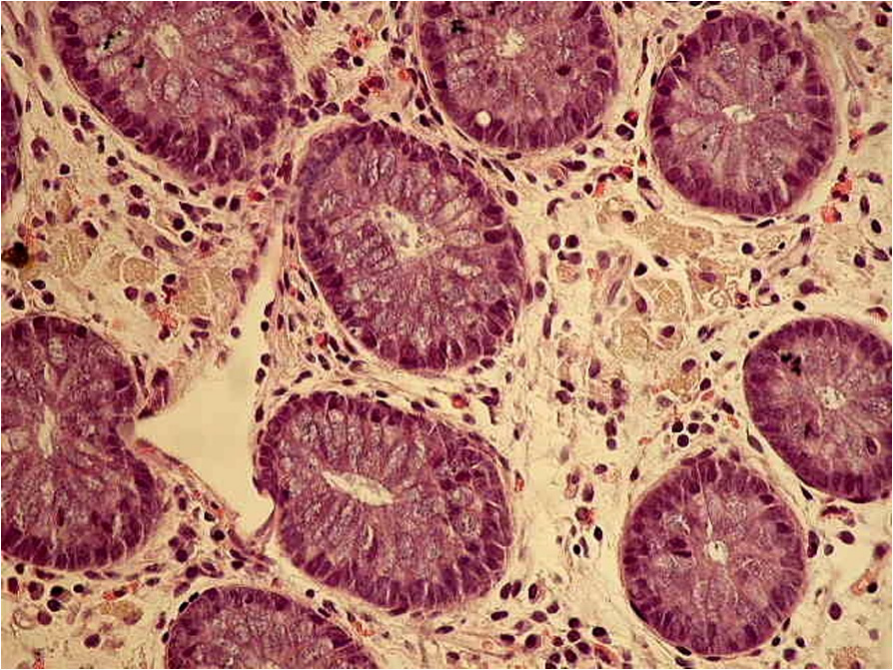
Presence of large pigment-laden histiocytes.

A second pathologist found clusters of well-formed noncaseating granulomas within the lamina propria as well as the presence of large pigment-laden histiocytes, the characteristic that best distinguishes CGD-associated enterocolitis from CD (Figures 3 and 4) [4].

Based on clinical history, laboratory data, granulomas on histology, and cutaneous infection from *Serratia*, CGD was diagnosed. CYBB gene sequencing for gp91phox, a membrane-bound subunit of NADPH oxidase, ultimately confirmed the diagnosis of X-linked CGD [1,2,5].

The patient began long-term antibiotic prophylaxis with trimethoprim-sulfamethoxazole (6 mg/kg/d given in two doses), and antifungal prophylaxis with itraconazole (5 mg/kg/d), and had complete resolution of cutaneous lesions within 3 weeks. The patient’s inflammatory parameters, which had been constantly elevated during the preceding 3 years, normalized. He has been completely symptom-free for 6 months.

## Conclusions

Gastrointestinal manifestations are a common and recurring problem in CGD, especially in the X-linked form, either at diagnosis or later. A study by van den Berg et al. found that 48% of CGD patients had experienced at least one episode of gastrointestinal manifestation [6]. The abnormal inflammatory response causes exuberant and persistent tissue granuloma formation, sometimes leading to obstructive symptoms. Clinical signs of CGD, including failure to thrive, diarrhea, colitis, bowel obstruction, perianal ulcerations and fistulas, anemia, and hypoalbuminemia, are also commonly seen in CD [7]. In addition, ASCA positivity is reported in up to 30% of CGD patients [8]. Histology from intestinal biopsies in CGD, with inflammation and granuloma formation, closely resembles CD. The presence of pigment-laden histiocytes within the lamina propria is the distinctive feature of CGD-associated colitis [4].

CGD is a rare but important differential diagnosis of chronic inflammatory bowel disease in childhood. Our patient showed clinical, laboratory, and histological characteristics hardly distinguishable from CD. One interesting aspect of this case is the presence of a positive serological marker (ASCA). Positivity of ASCA and cutaneous lesions consistent with pyoderma gangrenosum were confounding factors. Ulceration and abscess formation of the skin and soft tissues is a common feature of infections in CGD, most frequently from *Staphylococcus aureus* and *Serratia marcescens*. Therefore, CGD should always be considered in patients with a history of such infections.

The presence of markers typically associated with Crohn’s disease in our patient suggests that inflammatory bowel disease and granulomatous colitis associated with immune deficiency together represent a spectrum of colonic disease mediated by immune dysfunction and common pathogenic features.

Treatment for CGD, as for other immune deficiencies, is focused primarily on prevention with prophylactic antibiotic and antifungal therapy and therapy for potentially life-threatening infections. Long-term prophylaxis with IFN-γ has not been found to significantly change the rate of total infections per patient-year. Protocols of continued intensive surveillance and monitoring compliance with anti-infective regimens can significantly improve the quality of life and long-term survival in patients with CGD [9]. Stem cell transplantation is curative [10].

Clinicians and pathologists need to be aware that CGD is a differential diagnosis of CD, especially, but not exclusively, when occurring in early life. This case illustrates the importance of high clinical suspicion for an alternative diagnosis of immune deficiency in cases of presumed inflammatory bowel disease and opportunistic infection. Evaluation for clues of CGD in the medical history and physical examination of pediatric patients with suspected CD is mandatory. Although CGD is not a contraindication against immunosuppressive therapy commonly used in CD, anti-TNF-alpha agents, in combination with other immunomodulators, might be dangerous for these patients. An accurate discrimination between these two causes of granulomatous inflammation should always be obtained.

## Consent

Written informed consent was obtained from the patient for publication of this Case report and any accompanying images. A copy of the written consent is available for review by the Editor of this journal.

## Abbreviations

CGD: Chronic granulomatous disease; CD: Crohn’s disease; ASCA: Antibodies against saccharomices cerevisiae.

## Competing interests

In the past five years authors have not received reimbursements, fees, funding, or salary from an organization that may in any way gain or lose financially from the publication of this manuscript. None organization financing this manuscript. Authors don’t hold any stocks or shares in an organization that may in any way gain or lose financially from the publication of this manuscript. Authors don’t hold or are currently applying for any patents relating to the content of the manuscript. Authors don’t received reimbursements, fees, funding, or salary from an organization that holds or has applied for patents relating to the content of the manuscript. Authors don’t have any other financial competing interests. Authors don’t have non-financial competing interests in relation to this manuscript.

## Authors’ contributions

DM, RG, MT: carried out immunologic examinations, conceived of the study and and has given final approval of the version to be published. BM, CF, CS: carried out gastroenterologic examinations and participated in its design and coordination and helped to draft the manuscript. DG: has made gastroscopy and colonoscopy and has made substantial contributions to conception and design, and acquisition of data, and analysis and interpretation of data. IM: has been involved in NBT Test and participated in the sequence alignment and drafted the manuscript. MA: carried out the report of the biopsy and participated in the sequence alignment and drafted the manuscript. All authors read and approved the manuscript.

## Pre-publication history

The pre-publication history for this paper can be accessed here:

http://www.biomedcentral.com/1471-2431/14/156/prepub
